# Conflict pressure cooker: Nurse managers’ conflict management experiences in a diverse South African workplace

**DOI:** 10.4102/hsag.v24i0.1128

**Published:** 2019-11-29

**Authors:** Angela Koesnell, Petra Bester, Christi Niesing

**Affiliations:** 1 Africa Unit for Transdisciplinary Health Research (AUTHeR), Faculty of Health Sciences, North-West University, Potchefstroom, South Africa

**Keywords:** healthy work environment, positive practice environment, workplace diversity, conflict management, nurse manager, medical military organisation

## Abstract

**Background:**

Nurse managers are central to conflict management and a healthy work environment. South Africa is one of the most diverse countries globally and workplace diversity is a reality in healthcare organisations. There is a gap in academic literature on conflict management by nurse managers in diverse workplaces in South Africa.

**Aim:**

This research aims to understand nurse managers’ experiences of conflict management within a diverse South African workplace (military hospital) in order to facilitate a healthy work environment.

**Setting:**

The context was a diverse, medical military organisation servicing all nine South African provinces. This military hospital employed staff of varying nationalities, catering to military and private patients, and functioned within a strict hierarchical structure.

**Methods:**

Purposive sampling was used. Thirteen unstructured, individual interviews were conducted based on a qualitative, phenomenological design. The interviews were followed by content analysis and five main themes emerged as a result.

**Results:**

A hierarchical, diverse organisational culture complicates conflict management. The ranking structure, resource shortages, intergenerational dynamics, poor communication and distrust cause conflict. Nurse managers experience conflict daily and are central to conflict management. As such, they have certain personal characteristics and display specific conflict management skills. Conflict management skills can be taught, but this requires an intra- to interpersonal process. A major challenge for the nursing profession today is the younger nurses who seem less passionate and nurse managers who are under more pressure than before.

**Conclusion:**

A medical military organisation presents an organisational culture that combined with diversity is predisposed to conflict, which endangers the work environment. Yet, both conflict and workplace diversity can, when managed correctly, enrich a healthcare organisation. Nurses and nurse managers will benefit from reflective conflict management training as an intra- to interpersonal process.

## Background and problem statement

South Africa is one of the most diverse countries in the world (Fasset 2013), implying diverse workplaces, where people of different ages, gender, sexual orientation, physical appearance, national origin, education and religion are employed (Encyclopedia of Business and Finance 2016). Workplace diversity can either be enriching or detrimental to the work environment (Guillaume, Monro & Marshall 2016). The current nursing workforce reflects this diversity as it comprises three generational cohorts – the baby boomers (born between 1946 and 1964), generation X (born between 1965 and 1980) and the millennials (born between 1981 and 1999) – challenging effective communication and workplace harmony (Hoole & Bonnema 2015; Leiter, Price & Spence Laschinger 2010). When considering that Africa is one of the most diverse continents and South Africa has 11 official languages, one can imagine the given workplace diversity within typical work environments, including the health sector.

A nurse manager is responsible for enabling cooperation despite generational differences (Hahn 2011) and plays a pivotal role in creating and facilitating a healthy work environment (Ganz, Wagner & Toren 2015). Healthy work environments (also referred to as positive practice environments) support the well-being of healthcare providers and contribute to motivated, well-performing pools of personnel who deliver high-quality care (International Collaborating Partners for the Positive Practice Environments Campaign 2008). A healthy work environment in this context refers to a healthcare environment where patient outcomes are met, organisational goals are achieved and a work and care environment is created which is safe, healing, humane and respectful of the rights, responsibilities, needs and contributions of all people – including patients, their families and nurses (American Association for Critical Care Nurses [AACN] 2016). According to the guidelines of the AACN (2016), there are six standards of a healthy work environment:

- nurses who are proficient in both communication and clinical skills
- team members who pursue and foster true collaboration
- effective decision-making evident in valued and committed partners in policy
- appropriate staffing to ensure a balance between patient needs and nurse competencies
- meaningful recognition of the value that nurses and others bring to the organisation
- authentic leadership where nurse leaders fully embrace the imperative of a healthy work environment, authentically live it and engage others in its achievement.

This article contemplates that a healthy work environment can exist despite complex, diverse workplaces. In addition, conflict is inevitable in healthcare organisations (Yufenyuy 2018). It forms part of the everyday social, organisational and professional nursing life (Meyer et al. 2011; Tillet & French 2012). Conflict occurs where two or more parties are aware of different needs or values, which can be perceived as being incompatible (Booyens 2011; Johansen 2012; Tillet & French 2012). Sources of conflict within a healthcare facility include individual and group-related causes, organisational causes, workplace diversity and cross-generational conflict (Meyer et al. 2011).

The nurse manager is central to managing a turbulent, ever-changing work environment (Al-Hamdan, Shukri & Anthony 2011); developing and implementing a healthy work environment (Twigg & McCullough 2014) and simultaneously managing conflict. Conflict management is a comprehensive process that entails (1) recognising the conflict, (2) determining the intensity, (3) evaluating the effects of the intensity, (4) determining appropriate intervention methods and (5) observing the results (Çınar & Kaban 2012). The nurse manager should be enabled to detect the initial warning signs of conflict and to implement conflict management (Mohamed & Yousef 2014). Conflict is also central to nursing, as it is an emotionally charged profession (Heris & Heris 2011). It is essential that nurse managers are competent with managing emotions and interpersonal conflict effectively, and this requires them to have emotional intelligence (EI) (Heris & Heris 2011; Mohamed & Yousef 2014; Veshki et al. 2012). EI is the ability to monitor one’s own and also others’ feelings and emotions, to discern between different emotions and to use this information to guide one’s thinking and actions, to perceive emotions, to assimilate emotion-related feelings and to understand the information of these emotions and manage them (Mohamed & Yousef 2014). Supervisors, such as nurse managers, with a high EI will use an integrating style (both parties find a creative solution to satisfy each other’s concerns) and/or a compromising style (both parties win some and lose some to reach consensus) (Mohamed & Yousef 2014). Searches using the keywords such as ‘nurse manager’, ‘conflict management’, ‘conflict resolution’, ‘work environment’, ‘workplace diversity’, and ‘generational diversity’ were accessed via EbscoHost, ScienceDirect, eJournals and Google Scholar. Despite a strong presence of publication on workplace diversity and conflict management in healthcare organisations within the global arena, South Africa-based literature was limited. In fact, the most appropriate journal article found was written by Cremer (1980). Cremer (1980) identified the following methods of conflict management in the South African nursing practice: avoidance, smoothing, domination/forcing, compromise/bargaining, problem-solving by confrontation and integration as a positive approach to strengthening the self-respect of the persons involved. This dearth of conflict management evidence within the new South Africa led the researcher to ask the question, ‘what are nurse managers’ experiences of conflict management and their conflict management skills within a South Africa-based healthcare organisation?’ This research aimed to obtain a clear understanding of nurse managers’ conflict management skills within a diverse workplace, which could help provide recommendations to foster a healthy work environment.

## Method

A qualitative, explorative, descriptive and phenomenological design (Brink, Van Der Walt & Van Rensburg 2012) was deployed to gain an in-depth understanding of nursing managers’ real and lived experiences of their conflict management skills. The research setting was a highly diverse, level 3 medical military organisation. The personnel are representatives of all the different indigenous South African cultures, ethnicities, languages and qualification levels. There are also foreign members of different social strata, including ‘very important people’ (VIPs), civilians, private and force-enrolled employees. This medical military organisation, consisting of 556 beds, is situated in Gauteng province and renders specialised healthcare across the borders of all nine South African provinces. The data collection process started once ethical clearance had been obtained from the Health Research Ethics Committee of the university (certificate number: NWU-0019 5-S1), followed by the consent of the ethics committee of the participating organisation and the permission of the hospital management, including the nursing service manager. Privacy was ensured by conducting interviews in a private office, whilst confidentiality was enhanced by replacing participants’ names with codes on the transcripts. Once transcripts were completed, recordings were deleted from the digital voice recorder. Participants were assured that all the data were presented and reported from one data pool and that no participant’s identity or the identity of the participating hospital would be revealed during the research process and reporting.

In an effort to prevent a possible clash of interest and bias because of the research theme, the researcher utilised an independent person as a mediator to identify and recruit prospective participants. This proved to be a daunting task because of a high patient turnover and nursing staff shortage. The mediator was a nursing professional from the participating hospital and was delegated by the management. For participant engagement, the researcher first met with the mediator to explain the research purpose prior to recruitment and availed herself for more information should prospective participants deemed it necessary. The researcher had no direct personal or professional relationship with the participants. Purposive sampling with strict inclusion criteria led to the participation of 13 nurse managers, which occurred until data saturation reached. The inclusion criteria were as follows: participants had to be employed in a management position at the selected organisation for at least 2 years, had to hold a post-basic registration with the South African Nursing Council in health service management and had to be fluent in English. There were no exclusion criteria.

The mediator approached prospective participants during working hours, shared the informed consent form and invited them to participate in the study. Participant recruitment was challenged by intense staff shortages. Although no participant refused participation, obtaining a mutual opportunity for interviews was difficult. Once the participants had been identified, unstructured, individual interviews (Botma et al. 2010; Brink, Van Der Walt & Van Rensburg 2012; Burns & Grove 2010) followed in lieu of the qualitative phenomenological design. Interviews were conducted by the researcher only in a private office on the hospital’s premises and started on completion of voluntary informed consent forms. The researcher was a MCur candidate in health service management, who held a national nursing diploma and a BCur (Educationis et Administrationis) degree. She was employed at a Tshwane-based nursing college during this research whilst delivering lectures on psychiatric nursing science to undergraduate senior student nurses. The researcher’s interviewing skills were closely monitored by her supervisor who held a PhD in Nursing and is a psychiatric nursing specialist and who first supported the researcher through role-play to conduct these research interviews, acknowledging the difference between research interviews and therapeutic interviews. The interviews built on only one open interview question as aligned with phenomenology, namely, ‘[*a*]s a nurse manager, tell me how you experience conflict management and your conflict management skills within your workplace….’ The researcher utilised various verbal and non-verbal communication skills, such as clarification, summarising and minimal verbal responses, which enabled in-depth exploration of the participants’ experiences, and each interview lasted for at least 1 hour. Although no repeated interviews were conducted, participants were informed during each interview that the researcher would return in case a deeper exploration was necessary. Digitally recorded interviews were transcribed by an independent transcriber and were not returned to participants for further check and data analysis was conducted according to the six steps as laid out by Tesch (1990) and Creswell (2009) to generate units of meaning and to develop a description of the essence of participants’ experience. Consensus on the codes and the interpretation thereof was reached with a co-coder and led to the formulation of 5 themes and 12 sub-themes. No computer-assisted software was utilised to support the data analysis process.

The four strategies to increase rigour as stated by Lincoln and Guba (1985) were applied. This included the epistemological standards of trustworthiness as truth value, applicability, consistency, neutrality and an additional standard of authenticity. Truth value was strengthened through credibility, which was obtained through prolonged engagement with the literature and within the field. There were regular feedback sessions between the researcher and her supervisors. The researcher remained neutral despite being faced with the realities of conflict within diversified workplaces herself because subjectivity leads to criticism in qualitative research. Transferability was improved by means of a rich description of the research methodology as an audit trail with detailed findings and field notes. Consistency was strengthened by exploring all available models, theories and frameworks of conflict in the general environment and also the healthcare environment. Confirmability was strengthened through reflexivity captured as field notes. The researcher made regular summaries of content and clarifications of uncertainties during the interview process. Authenticity was supported by unstructured, individual and in-depth interviews where the true real lived experiences of nurse managers with dissimilar demographic characteristics were explored.

### Ethical consideration

Ethical clearance was obtained from the Health Research Ethics Committee of the North-West University (ethics number NWU-00019 5-S1).

Permission was granted by the ethics committee of the participating medical military organisation. The nursing director acted as a gatekeeper and allocated a mediator who assisted in engaging and recruiting participants. Informed consent was obtained from all the participants to participate in the study. Precautionary measurements were identified to minimise the risk of emotional discomfort. Anonymity and confidentiality were maintained throughout the research process.

## Results

The demographic profile of the 13 participants is presented in Table 1.

**TABLE 1:** Demographic profile of participants (*N* = 13).

Variables	*N*	%
**Gender**		
Male	3	23
Female	10	77
Age		
35–44 years	2	15
45–54 years	10	77
55–64 years	1	1
**Education**		
Bachelor’s degree	7	54
Honours degree	1	1
Language		
Setswana	4	31
Sesotho	2	15
isiZulu	1	1
Pedi	1	1
Afrikaans	1	1
Venda	3	23
Ndebele	1	1
**Nationality/race**		
Black	12	92
White	1	1
**Management experience**		
0–5 years	6	46
6–10 years	4	31
11–15 years	3	23
**Hospital experience**		
6–10 years	3	23
11–15 years	4	31
16–20 years	3	23
21–25 years	3	23
**Department or unit**		
General	3	23
Paediatric	1	1
Intensive care	4	31
ER	1	1
Primary healthcare	2	15
Theatre	1	1
Mental health	1	1

Of the 13 participants, 10 were women and 3 were men. This ratio correlates with the current ratios within the nursing profession in South Africa, which has a female-dominant representation (South African Nursing Council [SANC] 2014). The majority of the participants were aged 45–54 years (SANC 2014) and represented seven South African cultural groups. The participating nurse managers spoke with reasonable experience as the average time of employment at this particular hospital was 15 years, of which most had been employed as a manager for 10 years and had been in the nursing profession for 20 years.

**FIGURE 1:**
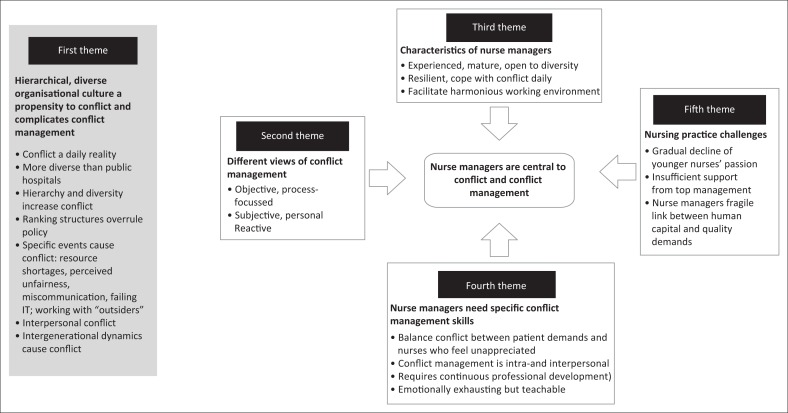
Depiction of the nurse managers’ experiences of conflict management within a South African military hospital.

Five main themes emerged from the in-depth interviews (see Figure 1). True to interpretive description, Figure 1 provides a summative outline of the findings and not a conflict management process. The over-arching themes were that nurse managers are central to conflict management within a diverse workplace; nurse managers had different experiences of conflict management and nurse managers believed that a hierarchical, inflexible, diverse organisational culture was prone to conflict. Specific characteristics for conflict management could be noted when the nurse managers presented with specific conflict management skills.

### Hierarchical, diverse organisational culture has a propensity to conflict and complicates conflict management

Within this rigid and diverse workplace, conflict presents as a daily reality. Some participants voiced that ‘conflict management is what is happening … on a daily basis’ and ‘conflict is there, we live with it on a daily basis’ (Participant 12). Participants experienced the medical military context as more diverse than the public health sector. ‘In my experience here, it’s quite different from the public sector …’ (Participant 6). The hierarchical, diverse organisational culture seems to brew the ‘perfect conflict storm’, complicating conflict management efforts. Hierarchy requires immediate response to patient and management demands, and this conflicts with the organisational limits such as availability of beds and staff. A nurse manager explained that ‘we learn about conflict management skills, but in the military environment when you have to apply it, the rank is the issue’ (Participant 4). Nurse managers explained that conflict within this medical military context increased when specific events occurred, namely, (1) resource shortages, (2) perceived unfairness in remuneration and promotions, (3) miscommunication and failing information communication technologies and (4) working with ‘outsiders’ such as civilians, private service-providers and non-military members. ‘It has really affected me so much [that] I wanted to resign’, said Participant 4, when speaking about the distrust and conflict surrounding staff promotions and an instance where an outsider entered the medical military organisation in a more senior position than employees who had been loyal to the force.

Ranking structures were seen to influence and over-rule policy. Nurse managers experienced the medical military organisation as an operations system within an unfair ranking system where higher ranked colleagues lacked effective conflict management skills. Therefore, nurse managers felt that their effective conflict management skills were wrongly demeaned within the ranking system. Subsequently, unresolved conflict increased nurses’ intent to leave. Within this organisational culture, nurse managers voiced the presence of distrust, especially in performance management.

Nurse managers described that the organisational culture permitted interpersonal conflict between colleagues and within multidisciplinary teams. These conflicts centred on perceived unfair promotions, which is best captured in the following words: ‘[*f*]rom top management to the last subordinate, there’s a lot of tensions between the interpersonal relationships’ (Participant 2). Interpersonal conflict also occurred in the absence of effective communication and because of perceived employee mediocrity and negative work attitudes. Furthermore, interpersonal conflict also arises from generational differences. Baby boomers experienced younger nurses as unteachable and disrespectful. One example highlighted during the interviews was that conflict rose when nursing students were absent over holiday seasons without notice, causing unplanned, increased work pressures. Participants made the following remarks in this regard:

‘Our nurses of this generation they don’t want to be told.’ (Participant 5)

‘We expect them to be responsible and take charge, but some of them compare themselves being young trend and they expect to be recognised better than the older, experienced trained personnel.’ (Participant 11)

### Different views on conflict management

Participants held different views on conflict management. One opined that ‘people are different and they manage differently’ (Participant 8). Participants experienced conflict management as objective (process-focused), subjective (personal) and reactive. Firstly, some nurse managers preferred an objective approach to conflict management as a step-by-step, mechanical process, discounting human dynamics: ‘I have stopped a number of times conflict issues where I felt that members started to get personal, because I don’t like any personal issues’ (Participant 4). Secondly, some nurse managers experienced conflict management as a subjective process. They valued being completely present during conflict management and spending sufficient time to resolve conflict on a personal level as effective conflict management skills. Thirdly, some nurse managers experienced conflict management as reactive. These managers proposed to not interfere at the first sign of conflict but to leave conflict to take its own course and to react later, as conflict management might be premature.

### Characteristics of nurse managers

Nurse managers identified characteristics within themselves that enabled them to manage conflict. Firstly, participants deemed it important that nurse managers should be experienced, mature and open to diversity. Nurse managers with this characteristic first seek to understand cultural differences and team members’ perspectives, and then take the necessary time to understand the actual events, knowing that lacking cultural awareness can complicate conflict management. ‘I was the only white member, a few times I had to stop a session to go and get more information to be able to assist’; ‘I constantly have to keep in mind the cultural differences when dealing with conflict’ (Participant 4). Secondly, participants explained that nurse managers should be resilient and able to cope with conflict daily. ‘… [*T*]he difficult part in the ward is conflict management’ (Participant 11). Thirdly, participants viewed the role of nurse managers as essential to facilitate a harmonious workplace by displaying an interest in and enabling continuous development of personnel through facilitative teamwork and deliberate team cohesion: ‘… you try to have some means of creating a harmonious environment in the department and be visibly hands-on and it’s less conflicts’ (Participant 5).

### Nurse managers need specific conflict management skills

In addition to certain characteristics, nurse managers described the need for specific conflict management skills within this conflict-prone organisation. Firstly, they need to balance attending to patient complaints and still let them feel that they are appreciated. Participants explained that certain levels of military patients display an entitlement, requesting immediate attention despite workplace demands. ‘… you’ll find they (the family) don’t even agree they just come down to the matrons’ office …’ (Participant 5). Secondly, nurse managers need to engage with conflict management from an intrapersonal to interpersonal level. Participants explained that nurse managers have to first know themselves, have to be experienced and also have the opportunity to grow personally and professionally. ‘I think that’s important growing from our experiences and learning from them in a human way’ (Participant 1). Thirdly, that nurses in general should participate in continuous professional development and self-growth opportunities to establish an environment where nurses can apply and reflect on their conflict-management skills during teaching–learning within the multi-disciplinary team before becoming managers. ‘… [*A*]s an operational manager we were suppose [*sic*] to be exposed or taken for a course (in conflict management) but it was not …’ (Participant 5). Fourthly, they need to cope with conflict management as an overwhelming experience but a teachable skill that can be taught and should be learnt.

### Nursing practice challenges

As the fifth theme, nurse managers’ experiences regarding nursing practice are highlighted. Participants experienced a decline in younger nurses’ passion for nursing and a lack of professional integrity and ownership amongst younger nurses as clear in the following statement: ‘… junior sisters lack a sense of responsibility because nobody wants to take the blame for mistakes …’ (Participant 2). Furthermore, nurse managers experienced the support from their supervisors in conflict management as insufficient:

‘If we working [*sic*] like a team, like from your top management to us area management to the unit managers at the unit level. It would be much easier to deal with conflict and more effectively.’ (Participant 1)

Nurse managers experienced being torn between human resources realities (overworked personnel struggling with work–life balance, absenteeism and staff shortages) and rendering quality care. The participants felt that quality care becomes compromised when nurse managers have to fulfil managerial duties whilst they also have to attend to basic nursing duties to keep up with the daily demands of the unit:

‘You are expected to do the nurse thing … expected to rinse the patient’s cup … fetch stock, at the end of the day it end up being the duty of the manager.’ (Participant 6)

## Discussion

Literature confirms the presence of conflict within a hierarchical culture and diverse workplace in general, but not specifically with regard to medical military organisations. Mazibuko and Govender (2017) confirmed conflict as prevalent in diverse South African workplaces and this was also an international norm. Conflict is a daily reality (Di Pietro & Di Virgilio 2013) faced by nurse managers (Guerra et al. 2011:364), especially within healthcare organisations and diverse workplaces, irrespective of them being military or not. Organisational culture impacts conflict frequency (Di Pietro & Di Virgilio 2013) in specialised healthcare organisations (Aitamaa et al. 2015). This especially holds true for an organisational culture that disregards existing problems associated with wrongs and corrective inertia. The organisational culture model proposed by Quinn and Gareth (cited in Hofstede 1991) confirms that a hierarchical culture aims to control and stabilise via rules, laws and execution of commands, and associates with competition, compromise, cooperation, consensus and avoidance conflict management styles (Ghorbani & Razavi 2011:716).

Various reasons for conflict in the workplace were confirmed. When considering that a controlled organisational culture (Tharp 2018:5) implies a formal workplace, governed by rules and being efficiency-minded, the conflict of a ranking system combined with a professional and caring nursing ethos is implicit. Resource shortages (Aitamaa et al. 2015), poor rewarding systems, poor teamwork (Di Pietro & Di Virgilio 2013), poor communication, a negative practice environment and distrust are among sources attributed to work-related conflict (Akpabio et al. 2015:106). Unresolved conflict impacts health outcomes (Arafat, Zaki & El-Kashif 2018:59, 64) and in turn affects the staff turnover. However, conflict in the context of working in a military medical organisation with non-military members could not be confirmed in the literature. Interpersonal conflict is associated with job dissatisfaction amongst nurses (Arafat et al. 2018:64), whilst ambiguous performance criteria can cause conflict (Omisore & Abiodun 2014:127) in the workplace. Finally, André (2018:13) confirmed the realities and challenges of intergenerational conflict in a generational diverse workplace in particular.

Regarding nurse managers’ different views on conflict management, Sudhakar (2015:204–231) presents it as a process with interdependent but sequential steps, whilst Bramsen and Poder (2018:2–14) explain the emotional realities of conflict management and Başoģul and Özgür (2016:228) urge the importance of EI in conflict management. A reactive view of conflict management was not confirmed in the literature as the ideal is to enable swift conflict resolution (Myatt 2012). Additionally, for nurse managers to engage in conflict management from an intrapersonal to an interpersonal level was not confirmed in the literature.

Concerning specific nurse managers’ characteristics, Oore, LeBlanc and Leiter (2015:301–309) explained that cognitive flexibility, a balanced focus on self and others, emotional regulation and a person–conflict fit are individual factors to enhance a positive reaction to workplace conflict. Obtaining EI and conflict management training are essential to enhance effective conflict management within healthcare organisations (Başoģul & Özgür 2016:228).

Attending to patients’ needs, striving for service excellence and maintaining a balanced nurse workload (Van Den Oetelaar et al. 2016) are described as pursuing different goals simultaneously, which can be argued as predisposing conflict in the workplace. Considering conflict activation through patient and family demands, Aitamaa et al. (2015) linked patient care-related demands to conflict between patients and staff, between nurses and doctors, between patients and their relatives. Nurse managers faced dissatisfied patients and their relatives, who had unrealistic demands and special requests from the nurses. A study in Nigeria confirmed that nurses preferred not to confront demanding patients and their family directly (Okhakhu, Okhakhu & Okhakhu 2014), although they experienced resentment when complying with doctors’ orders, which led to interpersonal and professional conflicts-in-care (Okhakhu et al. 2014). Nurse managers voiced the devaluing of the nursing profession and a negative public image (Aitamaa et al. 2015) as well as the lack of independent decision-making and associated authority related to nursing issues, which impact negatively nursing outcomes (Kieft et al. 2014).

Regarding nurse managers’ experiences that are nursing practice specific, certain nursing challenges presented as an international norm. Twibell and St Pierre (2012) highlighted that new nurses tend to leave their workplace because of heavy workloads and the inability to ensure patient safety, whilst nurses in the United Kingdom feel undervalued and underpaid (Anonymous 2018). Literature did not confirm nurse managers’ experiences that younger nurses lack professional integrity and ownership and not lack of supervisory support in conflict management. Rankin et al. (2015) confirmed the struggle to accomplish leadership goals whilst attending to nurse’s tasks as well.

## Conclusion

Nurse managers experience conflict daily as they are central within a diverse and controlled healthcare organisation. They should therefore be equipped to embrace workplace diversity, be enabled to manage conflict and be actively involved within nursing teams to create a harmonious working environment. Workplace diversity and conflict are exacerbated in a complex, hierarchal and fixed organisational culture, especially within medical military organisations. A ranking system, intergenerational dynamics, resource limitations and ineffective communication lead to conflict, increased distrust and perceived unfairness. Therefore, nurse managers require conflict management skills that are embedded within their intrapersonal, interpersonal and professional domains. The managers should know themselves, understand when a conflict is objective, subjective or reactive, and be self-aware when personal and professional values clash with organisational values. The nurse managers who participated in this study are experiencing a change in the nursing profession in that they observe a gradual decreased passion amongst younger nurses. Practical conflict management skills transfer to younger and less experienced nurses (who might prefer to cope with conflict through humour and avoidance) as well as through mentors providing support within a non-threatening environment could assist nurse managers within a diverse workplace with conflict management interventions.
